# Protective Effect of Natural Phenolic Acids, Rosmarinic Acid and Salvianolic Acid, Against Diabetic Atherosclerosis

**DOI:** 10.3390/ijms27156625

**Published:** 2026-07-24

**Authors:** Yaeram Won, Hye Jung Kim

**Affiliations:** 1 Department of Pharmacology, College of Medicine, Institute of Health Sciences, Gyeongsang National University, Jinju 52727, Republic of Korea; yrwon01@gmail.com; 2 Department of Convergence Medical Science (BK21 Four), Gyeongsang National University, Jinju 52727, Republic of Korea

**Keywords:** cardiovascular disease, endothelial dysfunction, phenolic acid, rosmarinic acid, salvianolic acid

## Abstract

Cardiovascular disease (CVDs) and atherosclerosis remain a major causes of morbidity and mortality worldwide. Endothelial dysfunction is a central driver of atherosclerosis, which is significantly accelerated in diabetes mellitus through hyperglycemia-induced oxidative stress, oxidized low-density lipoprotein (oxLDL) accumulation, and NLRP3 inflammasome activation. Phenolic acids, a class of plant-derived polyphenols, have gained attention as vasoprotective agents due to their antioxidant and anti-inflammatory properties. Among them, rosmarinic acid and salvianolic acid have gained attention, yet no previous review has systematically compared their distinct molecular mechanisms. This review addresses the gap by summarizing the pathophysiological mechanisms linking endothelial dysfunction to atherosclerotic progression and comparing how rosmarinic acid and salvianolic acid modulate oxidative stress, nitric oxide signaling, inflammatory responses, and NLRP3 inflammasome activation. Rosmarinic acid primarily exerts its effects through the p38 MAPK-FOXO1-TXNIP and AMPK/eNOS pathways, while salvianolic acid mainly acts via the PKM2/PKR and NF-ĸB/NLRP3 signaling pathways. Despite these findings, clinical evidence remains limited, primarily limited to Phase 1 pharmacokinetic studies and mixed-extract trials, leaving compound-specific efficacy in humans unverified. This review highlights these translational gaps and suggests prioritizing clinical trials of purified compounds, bioavailability optimization, and dose-standardization studies to advance rosmarinic acid and salvianolic acid toward clinical application in diabetic atherosclerosis.

## 1. Introduction

CVD is a group of disorders of the heart and blood vessels with high morbidity and mortality worldwide [1]. Hypertension, smoking, poor diet, and diabetes are the risk factors most closely related to the prevalence and incidence of CVD [2]. The total CVD prevalence was 271 million in 1990 and increased to 523 million in 2019. In addition, mortality was 12.1 million in 1990 and reached 18.6 million in 2019 [3]. Based on these reports, the prevalence of CVD from 2025 to 2050 is expected to increase from 598 million to 1.14 billion. Mortality is also expected to increase from 20.5 million to 35.6 million in 2050 [4].

Atherosclerosis, one of the leading causes of CVD, is a chronic inflammatory disease of the arterial wall that is fundamentally initiated and driven by endothelial dysfunction [5]. Endothelial dysfunction induces endothelial-to-mesenchymal transition (EndMT), vascular muscle cell proliferation, and plaque formation and instability, finally resulting in cardiovascular diseases such as atherosclerosis [6]. These pathological processes are known to worsen in individuals with diabetes, where metabolic disturbances further promote plaque development.

Diabetes mellitus is defined as a metabolic disease cluster characterized by elevated blood glucose levels due to impaired insulin production, reduced insulin effectiveness, or a combination of both [7]. Diabetes mellitus can be classified into type 1 and type 2. Both type 1 and type 2 can cause diabetes-associated atherosclerosis through hyperglycemia, inflammation, oxidative stress, and metabolic disturbances [8]. Diabetes mellitus is known to accelerate endothelial dysfunction by increasing ROS production, inflammation, oxLDL, and insulin resistance, resulting in severe atherosclerosis [9]. Accordingly, a growing body of research has focused on phytomedicine due to its ability to modulate oxidative stress and inflammation associated with diabetic atherosclerosis.

Phytomedicines consist of complex mixtures of plant metabolites that contain pharmacologically active compounds with therapeutic relevance. These plant-derived phytomedicines have been used as dietary supplements for human health, including cardiovascular health [10]. Among various phytomedicines, polyphenol secondary metabolites found in plants have been widely used in order to defend against pathogens and oxidative stress [11]. Polyphenols such as flavonoids, phenolic acids, tannins, and lignans are known to exhibit anti-inflammatory, antioxidant, antimicrobial, and antibiotic effects [12]. Phenolic acids, a class of polyphenols, show potential benefits in cardioprotective properties such as anti-hypertensive, anti-hyperlipidemia, and anti-hypertrophy [13]. These potential benefits have prompted extensive research into their therapeutic relevance for CVD.

Several reviews have addressed the cardioprotective roles of phenolic acids or related natural compounds against atherosclerosis. However, most of these reviews either examine phenolic acids broadly across cardiovascular disease without specifically addressing the diabetic context, in which hyperglycemia-driven oxidative stress and NLRP3 inflammasome activation substantially accelerate disease progression, or focus on a single compound in isolation. For instance, a recent preclinical systematic review and meta-analysis summarized the anti-atherosclerotic effects of salvianolic acid B alone, without extending the analysis to rosmarinic acid or to diabetes-specific pathological mechanisms. To date, no review has directly compared the molecular mechanisms of rosmarinic acid and salvianolic acid, two structurally related but mechanistically distinct hydroxycinnamic acid derivatives, within the specific context of diabetic atherosclerosis. Given that these two compounds converge on overlapping yet non-identical signaling pathways—including NLRP3 inflammasome regulation, eNOS/AMPK signaling, and pyrotosis—a comparative and mechanism-focused review is needed to clarify their distinct therapeutic potential and to guide future compound-specific clinical development.

In this review, we provide an overview of the pathophysiological and molecular mechanisms that lead to endothelial dysfunction and consequently to the initiation and progression of atherosclerosis. The review also highlights emerging therapeutic targets and summarizes recent findings related to potential interventions. Particular attention is given to the phenolic acids, especially focused on the comparison between rosmarinic acid and salvianolic acid B, which have recently gained interest for their potential to mitigate diabetic atherosclerosis.

## 2. Pathophysiology of Atherosclerotic Cardiovascular Diseases

### 2.1. Endothelial Dysfunction in Diabetic Atherosclerosis

Blood vessels consist of multiple cellular and structural components, including connective tissue, fibroblasts, endothelial cells, and vascular smooth muscle cells (VSMC). The endothelium, which is a single cell layer of endothelial cells, functions as a selectively permeable barrier separating the blood stream and the blood vessel wall [14]. The endothelial barrier plays a key role in various systemic functions, such as regulating vascular tone, maintaining fluid balance, and mediating host defense mechanisms [15]. Under physiological conditions, endothelial cells (ECs) maintain vascular homeostasis by balancing vasodilatory and vasoconstrictive factors, regulating blood flow with VSMCs, and producing vasodilators such as NO and prostacyclin I2 (PGI2) in response to tissue oxygen levels and metabolic demands [16]. In addition, ECs release a growth factor called vascular endothelial growth factor (VEGF), which exerts anti-apoptotic effects and regulates vascular permeability and endothelial cell migration [17]. Furthermore, ECs release and regulate cytokines and chemokines to control oxidative stress, vascular tone, inflammation, and thrombosis [18]. However, in pathological states, the endothelium adopts a phenotype that promotes oxidative damage, vasoconstriction, inflammation, and thrombogenesis, leading to endothelial dysfunction [19]. Endothelial dysfunction is mostly characterized by oxidative stress caused by ROS, reactive nitrogen species (RNS) or oxLDL [20].

### 2.2. Role of Oxidative Stress by RNS and ROS on Endothelial Dysfunction

**Figure 1:**
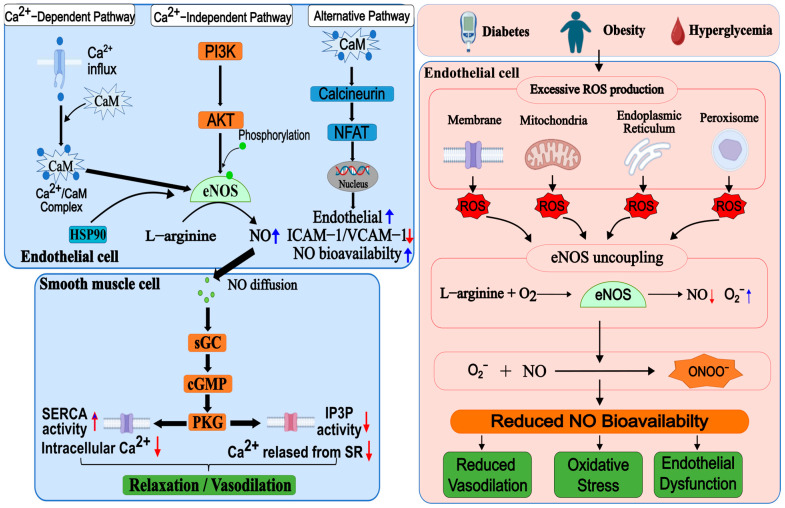
eNOS activation pathways under physiological conditions and eNOS uncoupling-induced endothelial dysfunction under pathological conditions. Under physiological conditions, eNOS is activated through Ca^2+^-dependent (CaM/Hsp90-mediated) and Ca^2+^-independent (PI3K/Akt-mediated Ser1177 phosphorylation) pathways to produce NO, which activates the sGC-cGMP-PKG cascade to promote vascular smooth muscle relaxation. Under pathological conditions such as hyperBglycemia, eNOS becomes uncoupled, resulting in diminished NO production and increased generation of superoxide (O_2_^−^), which reacts with NO to form peroxynitrite (ONOO^−^) and further reduce NO bioavailability, thereby driving endothelial dysfunction. CaM, calmodulin cGMP, cyclic guanosine monophosphate; eNOS, endothelial nitric oxide synthase; ER, endoplasmic reticulum; Hsp90, heat shock protein 90; NO, nitric oxide; ONOO^−^, peroxynitrite; O_2_^−^, superoxide; PI3K, phosphoinositide 3-kinase; PKG, protein kinase G; ROS, reactive oxygen species; sGC, soluble guanylyl cyclase.

NO is synthesized through nitric oxide synthase (NOS), which regulates tissue blood flow, controls vascular remodeling, protects against platelet aggregation, and leukocyte adhesion in our body [21]. There are three isoforms of NOS: neuronal NOS (nNOS), inducible NOS (iNOS), and endothelial NOS (eNOS) [20]. Among these, eNOS with several cofactors such as heme, flavin adenine dinucleotide (FAD), flavin mononucleotide (FMN), and tetrahydrobiopterin (BH_4_) produces NO in ECs by converting L-arginine with oxygen and nicotinamide adenine dinucleotide phosphate (NADPH) [22,23]. eNOS can be activated through both Ca^2+^-dependent and Ca^2+^-independent mechanisms or can be activated in an alternative pathway (Figure 1). In the Ca^2+^-dependent pathway, Ca^2+^ influx promotes the formation of a Ca^2+^-calmodulin (CaM) complex, which allows eNOS to detach from the plasma membrane and translocate into the cytoplasm. Heat shock protein 90 (hsp90) facilitates eNOS dimerization, protects it from proteasomal degradation, and recruits protein kinase phosphatase A to activate eNOS to produce NO [24,25]. In the Ca^2+^-independent pathway, the phosphoinositide 3-kinase/protein kinase B (PI3K/Akt) pathway phosphorylates eNOS at Ser1177, enhancing NO production. Alternatively, Ca^2+^/CaM complex activates calcineurin, which dephosphorylates nuclear factor of activated T cell (NFAT), promoting its translocation into the nucleus, where it functions as a transcription factor and indirectly modulates eNOS signaling [25]. Once synthesized, NO stimulates soluble guanylyl cyclase (sGC) to produce cyclic guanosine monophosphate (cGMP), which then activates protein kinase G (PKG). PKG promotes sarco/endoplasmic reticulum calcium ATPase (SERCA) and inhibits inositol 1,4,5-trisphosphate receptor (IP_3_R) to prevent Ca^2+^ release from the sarcoplasmic reticulum (SR). These mechanisms reduce cytoplasmic calcium levels, leading to the activation of myosin light chain phosphatase (MLCP) and resulting in smooth muscle relaxation and vascular relaxation [26]. In physiological conditions, the balance between ROS formation and degradation regulates vascular function [27]. Most of the ROS, such as H_2_O_2,_ are produced in the plasma/nuclear membranes and in several organelles, such as mitochondria, endoplasmic reticulum (ER), or peroxisomes. However, factors such as diabetes and obesity can lead to excessive ROS production, leading to oxidative stress, endothelial cell damage, and ultimately endothelial dysfunction [28,29]. Oxidative stress due to hyperglycemia can uncouple eNOS, which can markedly reduce NO production while generating ROS, mostly O_2_^−^ [23]. These O_2_^−^ can react with NO to produce ONOO^−^, resulting in diminished NO bioavailability [30,31].

### 2.3. Role of oxLDL on Atherosclerosis Development and Progression

**Figure 2:**
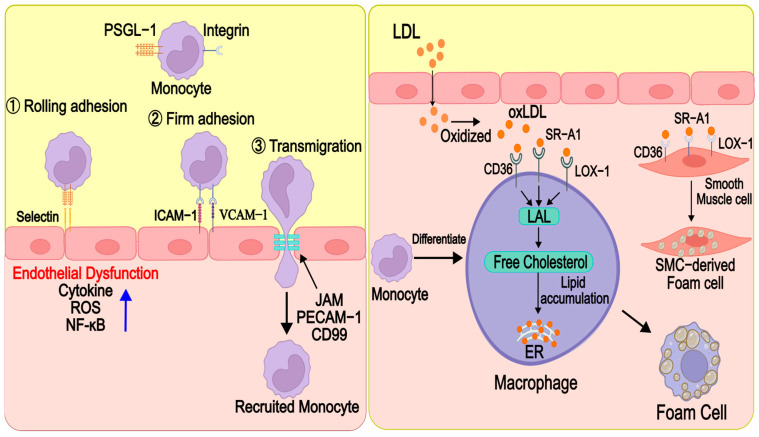
Monocyte recruitment, foam cell formation, and atherosclerotic plaque development in dysfunctional endothelium. Endothelial dysfunction induces expression of adhesion molecules (ICAM-1, VCAM-1, P-selectin), enabling monocyte rolling, firm adhesion, and transmigration into the subendothelial intima. Infiltrating monocytes differentiate into macrophages that internalize oxidized LDL via scavenger receptors (CD36, SR-A1, LOX-1); intracellular cholesteryl esters are hydrolyzed to free cholesterol and re-esterified by ACAT1, accumulating as lipid droplets that drive macrophage transformation into lipid-laden foam cells, an early hallmark of atherosclerotic plaque formation. CD36, cluster of differentiation 36; CD99, cluster of differentiation 99; ICAM-1, intercellu-lar adhesion molecule-1; JAM, junctional adhesion molecule; LAL, lysosomal acid lipase; LOX-1, lectin-like oxidized low-density lipoprotein receptor-1; LDL, low-density lipoprotein; oxLDL, oxidized low-density lipoprotein; PECAM-1, platelet endothelial cell adhesion molecule-1; PSGL-1, P-selectin glycoprotein ligand-1; SR-A1, scavenger receptor class A type 1; VCAM-1, vascular cell adhesion molecule-1.

Atherosclerosis begins in its early stages with the formation of foam cells, which play a key role in its progression [32]. During inflammation due to endothelial dysfunction, adhesion molecules such as ICAM-1 (Intercellular Cell Adhesion Molecule-1), VCAM-1 (Vascular Cell adhesion Molecule-1), and selectin (L-, P-, and E-selectin) are induced and expressed in ECs, facilitating the recruitment of circulating monocytes toward the subendothelial intima [33,34]. Initial adhesion of monocytes is mediated by selectin, mainly by P-selectin and its ligand, PSGL-1 (P-selectin glycoprotein ligand-1) (Figure 2) [35]. Under normal blood flow, the interaction of P-selectin and PSGL-1 is not sufficient to mediate binding. However, during inflammation, PSGL-1 on monocytes can bind to P-selectin on ECs, leading to weak and transient adhesion that slows monocyte movement and promotes rolling along the endothelial surface [35,36]. After weak adhesion, firm adhesion of monocytes to the endothelium is mediated by integrin interaction with ICAM-1 and VCAM-1 [37]. Then, monocytes are transmigrated through the EC junction [38]. Monocytes undergo polarization, a process marked by the reorganization of chemokine receptors and integrins toward the front of ECs. Thereafter, monocytes move toward the endothelial junction, where integrin-junctional adhesion molecule (JAM) interaction facilitate their passage through tight junctions. Finally, homophilic binding of platelet endothelial adhesion molecule 1 (PECAM-1) and cluster of differentiation 99 (CD99)enables monocyte transmigration into the intima [39].

LDL is a lipoprotein particle that transports cholesterol through the bloodstream [40]. When LDL enters the artery wall, it can be oxidized non-enzymatically or enzymatically by vascular cells [41]. Oxidized LDL plays an essential pathogenic role in the development and progression of atherosclerosis [42]. As monocytes differentiate to macrophages, these macrophages express scavenger receptors, including CD36, SR-A1, and lectin-like oxLDL receptor-1 (LOX-1). Oxidized LDL that is taken up by SR is trafficked to endosomes and lysosomes, where cholesteryl esters within the oxLDL are hydrolyzed into free cholesterol (FC) by lysosomal acid lipase (LAL) [43]. FC can then be re-esterified to form new cholesterol esters by acetyl-coenzyme A acetyltransferases 1 (ACAT1) and is ultimately stored as lipids in the ER as lipid droplets (LD). An excess accumulation of these newly formed cholesterol esters within the intima leads to the transformation of macrophages into foam cells, marking the early stage of atherosclerosis [43,44]. Additionally, smooth muscle cells also express SR and take up oxLDL, contributing to foam cell formation [45].

### 2.4. Role of NLRP3 Inflammasome Activation in Endothelial Dysfunction and Atherosclerosis Progression

Inflammasomes are intracellular protein complexes that play a key role in mediating immune responses to cellular damage and stress [46]. Inflammasomes consist of three types: pattern-recognition receptors (PRR) detecting pathogen-associated molecular patterns (PAMPs) or damage-associated molecular patterns (DAMPs), apoptosis-associated speck-like protein containing a caspase-recruitment domain (ASC), an adaptor sensor and effector, and caspase-1 activating interleukin 1β (IL-1β)/ interleukin-18 (IL-18), inducing pyroptosis [47]. Currently, five PRRs have been shown to form inflammasomes, and among these five, NLRP3 inflammasomes play a crucial role in various pathological conditions by acting as an intracellular sensor within the innate immune system [47].

**Figure 3:**
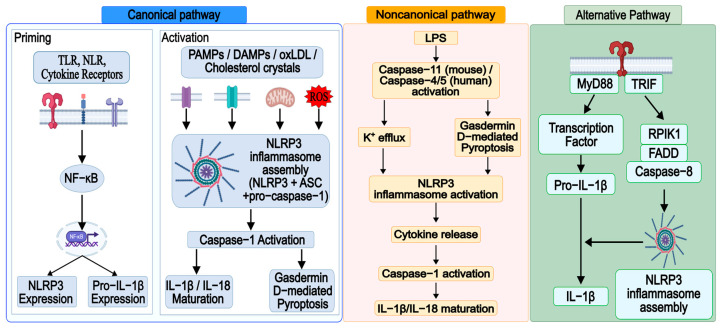
Canonical, noncanonical, and alternative pathways of NLRP3 inflammasome activation in vascular inflammation and atherosclerosis. In the canonical pathway, TLR/NLR-mediated priming upregulates NLRP3 and pro-IL-1β via NF-κB, followed by an activation step in which DAMPs/PAMPs trigger inflammasome assembly and caspase-1-dependent IL-1β/IL-18 maturation and pyroptosis. The noncanonical pathway is driven by LPS-activated caspase-11 (or caspase-4/5 in humans), while the alternative pathway proceeds through the TLR4–TRIF–RIPK1–FADD–caspase-8 axis independently of pyroptosis. Chronic activation of these pathways by oxLDL and cholesterol crystals sustains vascular inflammation and promotes atherosclerotic progression. DAMP, damage-associated molecular pattern; FADD, Fas-associated death domain; IL-1β, interleukin-1 beta; IL-18, interleukin-18; LPS, lipopolysaccharide; MyD88, myeloid differentiation primary response 88; NF-κB, nuclear factor kappa B; NLR, NOD-like receptor; NLRP3, NLR family pyrin domain containing 3; PAMP, pathogen-associated molecular pattern; RIPK1, receptor-interacting protein kinase 1; ROS, reactive oxygen species; oxLDL, oxidized low-density lipoprotein; TLR, Toll-like receptor; TRIF, TIR-domain-containing adapter-inducing interferon-β.

NLRP3 can be activated through three signaling pathways: canonical, noncanonical, and alternative activation of NLRP3 inflammasome (Figure 3) [48]. The canonical pathway requires two steps: the priming step and the activation step. During the priming step, Toll-Like Receptors (TLRs), cytokine receptors, or Nod-Like Receptors (NLRs) activate the NF-κB signaling pathway, which in turn upregulates the expression of NLRP3 and pro-IL-1β. During the activation step, PAMPs and DAMPs trigger NLRP3 inflammasome assembly through cellular events such as K^+^ and Cl^−^ efflux, mitochondrial dysfunction, and ROS [47,48]. Assembled inflammasome promotes caspase-1 activation, resulting in IL-1β and IL-18 maturation and gasdermin D-mediated pyroptosis [49]. The noncanonical pathway is triggered by LPS, which binds to caspase-11 in mice or caspase-4/5 in humans, leading to activation. Activated caspase-11 induces K^+^ efflux and gasdermin D-mediated pyroptosis and facilitates cytokine release [49,50]. The alternative pathway only requires TLR ligands to activate NLRP3 via the TLR4-TIR-domain-containing adaptor-inducing interferon (TRIF)-receptor-interacting serine/threonine-protein kinase 1 (RIPK1)-Fas-associated death domain protein (FADD)-caspase-8 axis, independent of K^+^ efflux and pyroptosis [49,50]. To sum up all the pathways, NLRP3 senses several stimuli such as DAMP, PAMP, oxLDL, and cholesterol, which leads to the formation of the NLRP3 inflammasome that activates caspase-1. Caspase-1 is involved in the maturation of pro-IL-1β/pro-IL-18 to IL-1β/IL-18, which amplifies vascular inflammation by inducing chemokine and adhesion molecule expression, thereby promoting monocyte recruitment [51]. While NLRP3 inflammasome activation is essential for host defense, its persistent activation in atherosclerosis is detrimental and contributes to disease progression. Unlike acute inflammatory responses, NLRP3 inflammasome activation in atherosclerosis becomes chronically engaged due to persistent metabolic signals. Cholesterol crystal-induced inflammasome activation promotes IL-1β release, which increases endothelial permeability and lipid accumulation, further enhancing crystal formation [52]. Additionally, inflammasome-driven pyroptosis releases intracellular signals that reactivate other macrophages, creating a self-amplifying inflammatory circuit [51]. In this regard, natural products, such as phenolic acids, that play a role in regulating endothelial dysfunction and chronic vascular inflammation without severe side effects, are emerging as potential therapeutic candidates for atherosclerosis.

## 3. Pharmacotherapeutic Potentials of Phenolic Acids on Diabetic Atherosclerosis

### 3.1. General Effect of Phenolic Acids on Human Health

**Figure 4:**
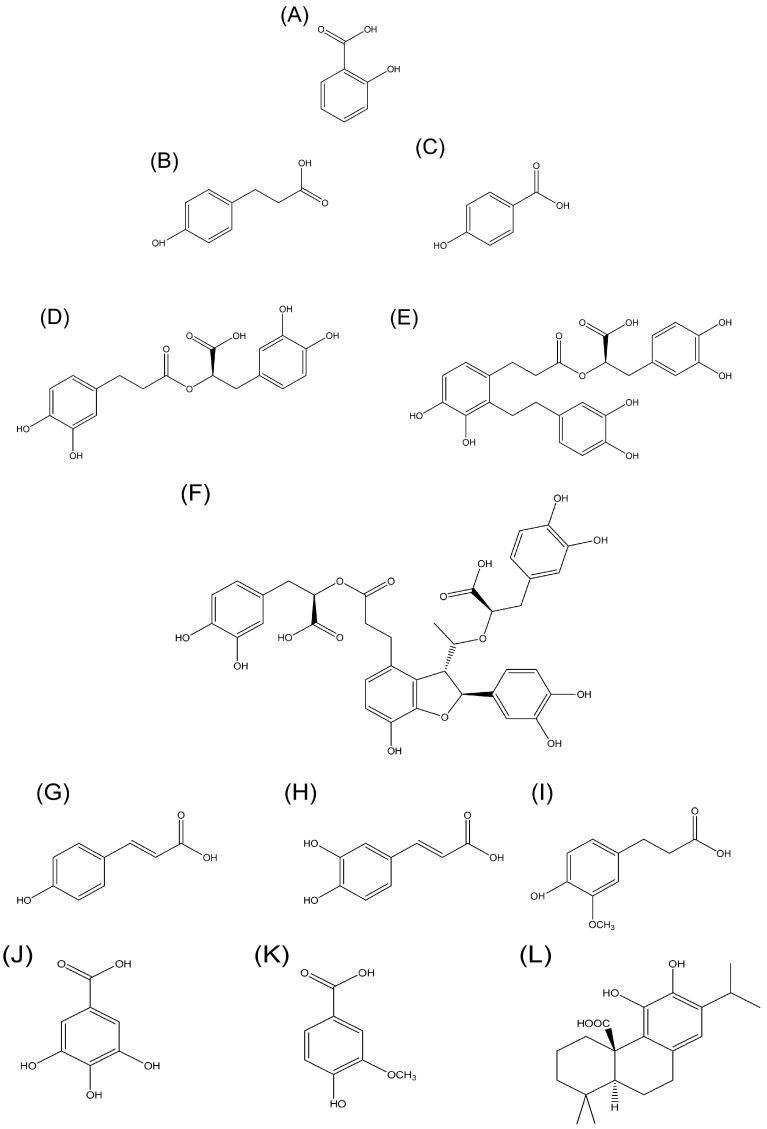
The chemical structure of phenolic acid subclasses and representative compounds discussed in this review. Phenolic acids are classified into hydroxycinnamic acids and hydroxybenzoic acids based on the position of the carboxyl group relative to the benzene rings, a structural distinction that underlies their differing antioxidant capacities. (**A**) Generic phenolic acid structure; (**B**) hydroxycinnamic acid backbone; (**C**) hydroxybenzoic acid backbone; (**D**) rosmarinic acid; (**E**) salvianolic acid A; (**F**) salvianolic acid B; (**G**) p-coumaric acid; (**H**) caffeic acid; (**I**) ferulic acid; (**J**) gallic acid; (**K**) vanillic acid; (**L**) carnosic acid.

Phenolic acids, secondary metabolites in plants, can be found in nuts and fruits. Phenolic acids have a phenol ring that possesses at least one carboxylic acid (Figure 4A) [53]. Phenolic acids possess various biological activities, such as antioxidant, antidiabetic, cardioprotective, anticancer, and anti-inflammatory effects [54]. Phenolic acids can be categorized into two subclasses based on their chemical structure [55]: hydroxycinnamic acid (Figure 4B) and hydroxybenzoic acid (Figure 4C) [13]. Hydroxycinnamic acid and hydroxybenzoic acid, derived from the non-phenolic group of cinnamic acid and benzoic acid, respectively, contain a hydroxylated benzene ring and a carboxyl group [13]. The main difference between these two groups lies in the position of the carboxyl group: hydroxybenzoic acids have a carboxyl group directly attached to the benzene ring, whereas hydroxycinnamic acids have a carboxyl group separated from the ring by a carbon–carbon double bond [56]. These structural differences show that hydroxycinnamic acid enhances antioxidant activity compared with hydroxybenzoic acid [57]. This is because the conjugated -CH=CH-COOH side chain of hydroxycinnamic acids promotes electron delocalization and stabilizes phenoxyl radicals more effectively than the structure of hydroxybenzoic acid [57]. The hydroxycinnamic acid group consists of compounds such as caffeic acid, ferulic acid, p-coumaric acid, and rosmarinic acid, while the hydroxybenzoic acid group includes gallic acid, vanillic acid, and carnosic acid [58].

### 3.2. Phenolic Acid

#### 3.2.1. Rosmarinic Acid

Rosmarinic acid (Figure 4D) is an ester of caffeic acid that is widely distributed in medicinal and culinary herbs [59]. Rosmarinic acid can be synthesized by two biosynthesis pathways in plants, which start from either L-phenylalanine or L-tyrosine to rosmarinic acid by rosmarinic acid synthase [60]. It possesses a broad spectrum of bioactivities, including antibacterial, anticancer, antioxidant, and anti-inflammatory effects [59]. Recent studies have also highlighted its potential roles in modulating insulin sensitivity, glucose homeostasis, and lipid metabolism [61]. Moreover, rosmarinic acid has been reported to attenuate oxidative damage by effectively scavenging ROS [62]. Under high glucose conditions, ROS production and thioredoxin-interacting protein (TXNIP) expression are increased through the p38 mitogen-activated protein kinase (p38 MAPK)-Forkhead box O1 (FOXO1) signaling axis in human aortic endothelial cells (HAECs). Rosmarinic acid dose-dependently reduced ROS production, attenuated NLRP3 inflammasome complex formation by inhibiting ASC upregulation/dimerization and caspase 1 activation by suppressing the p38-MAPK-FOXO1 signaling pathway and downregulating TXNIP expression [55,63]. Rosmarinic acid also dose-dependently increased the expression level of ATP-binding cassette transporter A1 (ABCA1) and ATP-binding cassette transporter G1 (ABCG1), cholesterol efflux transporters, thereby reducing foam cell formation and inflammasome activation by regulating p38 and extracellular signal-regulated kinases 1/2 (ERK1/2) pathway [64]. H_2_O_2_-treated HAEC downregulated the phosphorylation of AMP-activated protein kinase (AMPK) and eNOS. However, treatment with rosmarinic acid restored AMPK phosphorylation and eNOS levels, suggesting enhanced NO production, restoration of vasodilatory function, and attenuation of endothelial dysfunction [65]. Another study showed that high-glucose conditions with oxLDL treatment increased ICAM-1/VCAM-1 expression, monocyte-endothelial cell adhesion, Vascular endothelial cadherin (VE-cadherin) phosphorylation, endothelial permeability, and monocyte transendothelial migration in ECs. Rosmarinic acid attenuated oxLDL-induced ICAM-1/VCAM-1, monocyte-endothelial cell adhesion, and monocyte transmigration by suppressing the MAPK/FOXO1/TXNIP signaling pathway [66], in a dose-dependent manner. Also, rosmarinic acid effectively suppressed PDGF-BB-induced hyperproliferation and migration of VSMC, a key pathological process that drives neointimal formation and accelerated atherosclerotic plaque development by activating the kelch-like ECH-associated protein 1 (Keap1)-Nrf2 antioxidant pathway [67].

In vivo experiments also showed the protective effect of rosmarinic acid against atherosclerosis. When atherosclerosis-induced C57BL/6J male mice were treated with different doses of rosmarinic acid under high-fat conditions, rosmarinic acid decreased body weight, attenuated oxidative stress by upregulating superoxide dismutase (SOD) and glutathione peroxidase (GSH-Px) activity, and reduced lipid plaque formation in the vessels [68]. Another study in C57BL/6 mice under high-fat conditions showed that rosmarinic acid decreased body weight, increased hepatic LDL-R and SR-BI expression, and enhanced cholesterol excretion by upregulating ABCG5 and ABCG8 transporter expression. These results suggest that improvements in the lipid profile may contribute to a reduced risk of atherosclerosis [69]. Also, in a rat carotid artery balloon injury model, rosmarinic acid effectively inhibited ADP- and collagen-induced platelet aggregation, reduced intracellular calcium concentrations, and significantly reduced neointima hyperplasia and collagen deposition in response to vascular injury [67].

#### 3.2.2. Salvianolic Acid

Salvianolic acid A and B (SAA, SAB) (Figure 4E,F) are mostly found in *Salvia miltiorrhiza* (Danshen), a medicinal plant widely used in traditional Chinese medicine [70]. Both SAA and SAB, water-soluble metabolites, have been shown to exhibit various bioactivities, including anti-inflammatory, anticancer, and antioxidant effects [71]. As previously described for rosmarinic acid biosynthesis, salvianolic acid also originates from either L-phenylalanine or L-tyrosine via rosmarinic acid formation. However, the biosynthetic pathway from rosmarinic acid to salvianolic acid remains unclear [72]. Despite this uncertainty, both SAA and SAB have been investigated for their therapeutic effects on atherosclerosis, which include reducing endothelial dysfunction, inhibiting smooth muscle cell migration and proliferation, and blocking platelet aggregation [73].

Under high-glucose conditions, human umbilical vein endothelial cells (HUVECs) showed enhanced NLRP3 inflammasome activation, as evidenced by increased NLRP3-ASC complex formation, leading to pyroptosis and subsequent endothelial dysfunction. SAA treatment inhibited NLRP3 inflammasome activation and attenuated pyroptosis through suppression of the pyruvate kinase M2/protein kinase R (PKM2/PKR) pathway, a key regulator of NLRP3 inflammasome activation [74]. Tumor necrosis factor-alpha (TNF-α) stimulation activated ROS/ nuclear factor kappa B (NF-ĸB) signaling in HUVECs, leading to increased ICAM-1 expression, NLRP3 inflammasome activation, and pyroptosis-related responses, which collectively contributed to endothelial dysfunction. SAB treatment alleviated TNF-α-induced pyroptosis in HUVECs by reducing ROS production and inhibiting NF-ĸB/NLRP3 inflammasome signaling [75]. In endothelial progenitor cells (EPC), tunicamycin-induced ER stress triggered NLRP3 inflammasome activation and promoted pyroptosis. SAB suppressed these effects through modulation of the AMPK/FOXO4 and Syndecan-4/Rac1-related signaling, thereby attenuating inflammasome activation and pyroptotic cell death [76].

Several in vivo experiments also showed that SAA treatment at the dose of 20 mg/kg or 40 mg/kg significantly reduced atherosclerotic plaque formation, lipid accumulation, and inflammatory cytokine production in high-fat diet C57BL/6 and *ApoE*^−/−^ mouse models [77]. In the Zucker diabetic fatty rat model of atherosclerosis by a high-fat diet and vitamin D_3_, SAA treatment at various doses significantly reduced the expression of NLRP3 and cleaved caspase-1 and decreased NF-κB and IL-1β [78]. These findings suggest that SAA suppresses the activation of both the NLRP3 inflammasome and NF-κB pathway. In *db/db* C57/BL/KsJ mouse model, SAB treatment (10 mg/kg/day) significantly decreased ROS production and bone morphogenetic protein 4 (BMP4) expression via inhibition of the p38 MAPK/ JNK/caspase 3 pathway [79]. Consistently, in a high-fat diet-induced atherosclerotic mouse model of atherosclerosis, SAB treatment attenuated plaque formation, reduced ROS generation and apoptosis in the atherosclerotic lesion, and suppressed inflammatory responses through inhibition of the NF-κB signaling and subsequent NLRP3 inflammasome activation [75].

### 3.3. Other Phenolic Acids

p-Coumaric acid (Figure 4G), a hydroxycinnamic acid derivative biosynthesized from phenylalanine and tyrosine through the shikimate pathway, has been reported to possess antioxidant, antitumor, and antibacterial activities [80]. In THP1-derived macrophages co-stimulated with oxLDL and LPS, foam cell formation, inflammatory cytokine production, and NF-κB activation were markedly increased. Treatment with p-coumaric acid dose-dependently inhibited these responses, suggesting its potential protective effect against atherosclerosis [81]. In a high-fat/high-sucrose diet-induced metabolic disorder model using C57BL/6J mice, administration of p-coumaric acid (50 mg/kg) suppressed the priming and activation of the NLRP3 inflammasome through inhibition of TLR4/NF-κB signaling, ER stress, and oxidative stress, thereby attenuating caspase-1-mediated IL-1β secretion [82].

Caffeic acid (Figure 4H), a polyphenolic compound belonging to the hydroxycinnamic acid family, has been studied for antioxidant properties and has demonstrated protective effects against inflammation, cancer, and neurodegenerative diseases [83]. In oxidative stress-induced HUVECs, low concentrations of caffeic acid increased NO bioavailability, enhanced ECs proliferation, and promoted angiogenic activity, suggesting its endothelial protective effects [84]. Caffeic acid methyl ester, an ester derivative of caffeic acid, is also known for its anti-inflammatory and anti-allergic properties. In LPS-induced HUVECs, CAME treatment dose-dependently reduced cytokine secretion as well as COX-2 and iNOS expression through activation of the heme oxygenase-1 (HO-1)/Nrf2 antioxidant pathway and suppression of aberrant NF-κB signaling [85]. In vivo studies, administration of caffeic acid (20 mg/kg) to *ApoE*^−/−^ mice attenuated atherosclerotic lesions by enhancing cholesterol efflux through upregulation of ABCA1 and ABCG1 expression [86]. Furthermore, in a photochemically induced cerebral thrombosis model using C57BL/6J mice, pretreatment with caffeic acid (1.25 or 5 mg/kg) significantly reduced platelet accumulation and delayed both thrombus formation and vascular occlusion. Similarly, in an ADP-induced cerebral venule thrombosis model, pretreatment with caffeic acid (5 mg/kg) reduced platelet adhesion and inhibited thrombus formation [87].

Ferulic acid (Figure 4I), a hydroxycinnamic acid derivative biosynthesized from caffeic acid, has been widely recognized for its antioxidant, antidiabetic, and antihypertensive properties [88,89]. TNF-α-stimulated HUVECs exhibited reduced NO production, causing endothelial dysfunction. Treatment with ferulic acid restored arginosuccinate synthase expression, a key enzyme involved in the regeneration of L-arginine required for NO synthesis, and suppressed NF-κB signaling, thereby alleviating inflammation-induced endothelial dysfunction [90]. In HUVECs exposed to advanced glycation end products, ferulic acid dose-dependently attenuated AGEs-induced endothelial inflammation by reducing intracellular ROS generation and suppressing the expression of ICAM-1, VCAM-1, and NLRP3 through modulation of the p38 MAPK and NF-ĸB signaling pathways [91]. In addition, in PDGF-induced VSMCs, ferulic acid inhibited cell proliferation, induced G1-phase cell-cycle arrest, and increased p21 expression in a dose-dependent manner via activation of the PI3K/AKT/eNOS signaling pathway [92]. For in vivo high-fat diet models, ferulic acid treatment significantly reduced total cholesterol (TC), triglyceride (TG), and LDL-C levels and attenuated plaque formation by modulating lipid metabolism, including upregulation of AMPK and downregulation of sterol regulatory element-binding protein 1 (SREBP1), acetyl-CoA carboxylase 1 (ACC1), and stearoyl-CoA desaturase 1 (SCD1) expression [93].

Gallic acid (Figure 4J), a naturally occurring hydroxybenzoic acid derivative widely distributed in plants, has been extensively studied for its antioxidant, antimicrobial, anti-inflammatory, and anticancer properties [94]. In LPS-primed macrophages, gallic acid suppressed ROS generation and inhibited NLRP3 inflammasome activation and pyroptosis in an Nrf2-dependent manner, suggesting that it may indirectly contribute to the attenuation of endothelial dysfunction by reducing macrophage-mediated inflammatory responses [95]. For an in vivo study using C57BL/6 male mice, angiotensin II infusion for 2 weeks significantly increased systolic blood pressure, promoted eNOS protein degradation, and enhanced ROS generation, leading to endothelial dysfunction and hypertension. Oral administration of gallic acid prevented eNOS degradation, restored NO production, and improved endothelial dysfunction [96]. In another study, male and female *ApoE*^−/−^ mice model treated with 0.2% of gallic acid in drinking water, gallic acid reduced plaque formation and inflammation in male mice but not in female mice, suggesting a sex-dependent anti-atherosclerotic effect [97]. However, in the same mice model, a low dose of gallic acid (20 mg/kg) failed to reduce hyperlipidemia, hepatosteatosis, adipogenesis, or atherogenesis, indicating that higher doses or prolonged treatment are required to achieve therapeutic efficacy [98].

Vanillic acid (Figure 4K), a naturally occurring phenolic acid widely distributed in plants, has been reported to possess anticancer, anti-obesity, antidiabetic, antibacterial, anti-inflammatory, and antioxidant effects [99]. TNF-α-stimulated HUVECs reduced cell viability, increased apoptosis, reduced the expression of eNOS, and NO production, but treatment with vanillic acid reversed these effects by activating the Akt-eNOS signaling pathway, thereby enhancing antioxidant defense and improving endothelial function [100]. In an in vivo model of nitric oxide-deficient hypertension induced by L-NAME (NG-nitro-L-arginine methyl ester), treatment of vanillic acid (50 mg/kg) normalized elevated lipid metabolism including TC, TG, free fatty acid (FFA), and phospholipid (PL), and restored eNOS expression. These findings suggest that vanillic acid mitigates NO-deficiency-driven vascular and metabolic dysfunction [101].

Carnosic acid (Figure 4L) is a phenolic diterpenoid containing two phenolic hydroxyl groups and a carboxyl group, which can be oxidized to form carnosol [102]. It has been widely reported to possess antioxidant and antimicrobial properties [103]. Most recent studies investigating the vascular effects of carnosic acid have been conducted in retinal ECs. In these cells, carnosic acid dose-dependently reduced iNOS expression, NO production, and the levels of pro-inflammatory cytokines, including IL-6 and TNF-α, by enhancing SIRT1 (sirtuin1), an enzyme that promotes mitochondrial biogenesis, enhances oxygen utilization, and activates AMP-activated protein kinase lowering the damage of diabetic retinopathy. Similarly, in vivo studies have shown that treatment with carnosic acid (50 mg/kg) restored the SIRT1/p53/SLC7A11 pathway, which protected retinal neuron damage, preserved retinal microvascular and endothelial function in diabetic rats, compared with untreated diabetic rats [104]. The experimental models, major mechanisms, and reported effects of the phenolic acid discussed above are summarized in Table 1.

### 3.4. Clinical Translation and Limitations

Despite extensive preclinical evidence supporting the anti-atherosclerotic potential of rosmarinic acid and salvianolic acid, direct clinical validation is still limited. There are currently no randomized controlled trials specifically evaluating the effects of isolated rosmarinic acid or SAA and SAB on endothelial function or cardiovascular outcomes. Additionally, the available human data primarily come from studies using rosemary extract, which contains a mixture of various phenolic compounds rather than purified rosmarinic acid. one study reported that oral supplementation with rosemary extract containing 10.30 mg of rosmarinic acid improved flow-mediated dilation of the brachial artery and decreased plasma PAI-1 activity in healthy young adults [105]. Another pilot randomized, double-blinded, placebo-controlled clinical trial using rosemary and ginger extracts showed a significant reduction in oxidative DNA damage in low dose and increased erythrocyte phosphorylated eNOS expression at high dose suggesting that rosmarinic acid can modulate endothelial-related signaling in humans [106]. For SAA, available cardiovascular-related human studies have mainly used blood samples from patients with type 2 diabetes mellitus or acute coronary syndrome [107,108]. In these studies, platelets were isolated from patient blood samples and treated with SAA ex vivo to evaluate platelet aggregation and activation. SAA treatment reduced ADP- or thrombin-induced platelet aggregation and decreased the expression platelet activation. Also, a recent study reported that salvianolate for injection (SFI), a SAB-rich formulation derived from *Salvia miltiorrhiza* significantly reduced major cardiac events and improved endothelial function-related parameters in patients with acute myocardial infarction [109].

Moreover, a major pharmacokinetic barrier further complicates clinical translation of rosmarinic acid: it has extremely low oral bioavailability, generally below 10%. This low bioavailability is primarily due to its poor lipophilicity, extensive metabolism by intestinal microflora, and hepatic first-pass metabolism [110]. Similar bioavailability issues have been reported for salvianolic acids, as their water-soluble depside structure restricts membrane permeability and limits systemic exposure after oral administration [111]. These pharmacokinetic constraints imply that plasma concentrations achievable through oral intake in human may fall well below the level considered effective in vitro, raising uncertainty about the potential for replicating preclinical efficacy at clinically feasible doses. Various strategies, including nanoparticle-based delivery, liposomal encapsulation, and structural derivatization, have been investigated to enhance the bioavailability of both compounds [112,113,114,115]. However, none of these approaches has yet progressed to clinical evaluation in the context of atherosclerosis or endothelial dysfunction. Taken together, while preclinical findings support a mechanistic rationale for the use of rosmarininc acid and salvianolic acid in diabetic atherosclerosis, their clinical translation is currently constrained by the absence of compound-specific efficacy trials, reliance on mixed-extract formulations in existing human studies, and unresolved bioavailability limitations that must be addressed through formulation optimization before clinical development can proceed.

## 4. Discussion

Phenolic acids represent a major group of dietary bioactive compounds with potential cardioprotective effects. Several studies suggest that phenolic acids can attenuate cardiovascular disease progression driven by pathological factors such as oxidative stress, inflammation, endothelial dysfunction, and plaque formation. These effects are associated with the regulation of cellular defense systems, including enhanced eNOS activity, increased NO bioavailability, and activation of antioxidant pathways.

Importantly, phenolic acids commonly share antioxidant and anti-inflammatory properties but may also regulate cardiovascular protection through different molecular pathways. For example, rosmarinic acid and salvianolic acid have been shown to inhibit NLRP3 inflammasome activation, although their regulatory mechanisms may differ. Similarly, caffeic acid and gallic acid show antioxidant and anti-inflammatory effects through activation of the Nrf2 pathway, whereas ferulic acid may reduce vascular inflammation and oxidative stress by suppressing NF-ĸB signaling. Vanillic acid may improve endothelial function by enhancing Akt-eNOS-mediated NO bioavailability. Taken together, these findings suggest that each phenolic acid subtype prevents or attenuates cardiovascular disease through distinct mechanisms.

Despite these promising preclinical findings, clinical evidence supporting the cardiovascular benefits of phenolic acids remains insufficient. Some human intervention studies using phenolic acid-rich foods or enhanced phenolic acid bioavailability have reported improvement in vascular function. For example, randomized, double-blind, placebo-controlled clinical studies show that a 500 mg capsule of ferulic acid twice a day for 6 weeks improved lipid profile, including TG, TC, and LDL-C, and reduced oxidative stress and inflammatory markers, suggesting that ferulic acid may help attenuate CVD-related inflammatory and oxidative stress [116]. However, since these interventions involved mixed extracts containing multiple phenolic compounds, further clinical studies using purified rosmarinic acid are needed to determine its specific effects on endothelial function and CVD. However, as these studies did not directly evaluate endothelial function or CVD outcomes, further clinical studies are needed to clarify their effects on CVD.

A thorough evaluation of the current evidence base is essential, going beyond mechanistic and preclinical findings, most studies reviewed here rely on rodent or cell-culture models using isolated rosmarninc acid or salvianolic acid at doses that often exceed physiologically achievable concentrations in humans. In addition, there is a lack of direct head-to-head comparisons between the two compounds. Reproducibility across studies is also challenging to evaluate because the experimental models (e.g., HUVEC vs. HAEC, high-glucose vs. oxLDL co-treatment, diet-induced vs. genetic atherosclerosis models) and dosing regimens vary significantly, which limits comparability across studies. Furthermore, most in vivo studies report short-term outcomes, and data on long-term efficacy or safety are currently unavailable.

From a regulatory perspective, neither rosmarinic acid nor salvianolic acid has been evaluated under a formal clinical development pathway for cardiovascular indications. Both compounds would likely be classified under the U.S. Food and Drug Administration’s botanical drug development framework, which requires well-characterized, standardized extracts and dose-ranging clinical trials to establish safety and efficacy prior to approval [117]. To date, no standardized clinical development program has been reported for either compound, representing a significant translational barrier that must be overcome before these phenolic acids can be considered for therapeutic application in diabetic atherosclerosis.

## 5. Conclusions

Endothelial dysfunction, driven by oxidative stress, oxLDL accumulation, and NLRP3 inflammasome activation, represents a central pathological axis linking diabetes mellitus to accelerated atherosclerosis. Among natural phenolic acids investigated for their vasoprotective potential, rosmarinic acid and salvianolic acid have emerged as particularly promising candidates. Rosmarinic acid primarily exerts its protective effects through suppression of the p38 MAPK-FOXO1-TXNIP axis and enhancement of AMPK/eNOS signaling, thereby reducing ROS generation, restoring NO bioavailability, and limiting monocyte-endothelial adhesion. SAA and SAB, in contrast, act predominantly through inhibition of the PKM2/PKR and NF-ĸB/NLRP3 signaling pathways, attenuating pyroptosis and inflammasome-driven vascular injury. Despite sharing antioxidant and anti-inflammatory properties, these mechanistic distinctions suggest that rosmarinic acid and salvianolic acid may offer complementary, rather than redundant, therapeutic value in diabetic atherosclerosis.

Nevertheless, substantial challenges remain before clinical application. The current evidence base is derived almost entirely from in vitro and rodent models, and direct clinical trials evaluating isolated rosmarinic acid or salvianolic acid on endothelial function or atherosclerotic outcomes are lacking; available human data are limited to Phase 1 pharmacokinetic and safety studies or to mixed-extract interventions that cannot isolate compound-specific effects. Furthermore, issues related to bioavailability, metabolic stability, optimal dosing, and long-term safety remain inadequately characterized, and manufacturing standardization for pharmaceutical-grade preparations has not been established. These translational gaps are compounded by methodological limitations evident across the preclinical literature reviewed here, including inconsistent dosing regimens and treatment duration, predominant reliance on short-term, male-only animal studies despite evidence of sex-dependent effects [118], and a lack of head-to-head comparison between rosmarinic acid and salvianolic acid within unified experimental systems. Addressing these gaps will require preclinical studies that adopt standardized, sex-inclusive designs with extended observation periods, alongside well-designed, placebo-controlled clinical trials using purified compounds to directly assess endothelial function and cardiovascular outcomes in diabetic populations. Parallel efforts to improve bioavailability, establish optimal dosing, and clarify subtype-specific molecular targets will be essential to guide rational combination strategies or derivative development.

## Figures and Tables

**Table 1:** Effects of phenolic acid derivatives on the pathophysiology of atherosclerotic cardiovascular diseases.

Hydroxycinnamic Acid				
Compound	Experimental Model	Mechanisms	Effects	Reference
Rosmarinic Acid	In vitro (HAEC)	↓ROS ↓TXNIP via p38 MAPK-FOXO1 ↓ASC/caspase-1	Suppresses NLRP3 inflammasome activation without altering NLRP3 expression	[55,63]
	In vitro (macrophage)	↑ ABCA1 ↑ ABCG1 via p38/ERK1/2	Reduces foam cell formation and enhances cholesterol efflux	[64]
	In vitro (HAEC)	↓p-AMPK ↓eNOS	Enhanced NO production, restored vasodilatory function, and attenuated endothelial dysfunction	[65]
	In vitro (monocyte)	↓MAPK/FOXO1/TXNIP pathway	Attenuated ICAM-1/VCAM-1, monocyte-endothelial cell adhesion, monocyte transmigration	[66]
	In vitro (VSMC)	↑ KEAP1-Nrf2 pathway	Inhibits VSMC proliferation and migration	[67]
	In vivo (C57BL/6J mice)	↓ Oxidative stress ↓ Lipid plaques	Reduces body weight and vascular lipid accumulation	[68]
	In vivo (C57BL/6J mice)	↑ LDL-R ↑ SR-BI ↑ ABCG5/8	Improves lipid metabolism and cholesterol excretion	[69]
	In vivo (Rat)	↓ Platelet aggregation ↓ Ca^2+^ influx	Attenuates neointimal hyperplasia and collagen deposition	[67]
Salvianolic Acid A	In vitro (HUVEC)	↓ PKM2/PKR pathway	Inhibited NLRP3 inflammasome, and attenuated pyroptosis	[74]
	In vivo (C57BL/6J mice, *ApoE*^−/−^)	↓ Lipid accumulation ↓ Cytokines	Reduces atherosclerotic plaque formation	[77]
	In vivo (Rat)	↓ NLRP3 ↓ Caspase-1 ↓ NF-ĸB ↓ IL-1β	Suppresses inflammasome activation	[78]
Salvianolic Acid B	In vitro (HUVEC)	↓ ROS ↓ NF-ĸB	Attenuated pyroptosis, and suppressed NLRP3 inflammasome	[75]
	In vitro (EPC)	↓ AMPK/FOXO4 ↓ Syndecan-4/Rac1-related signaling	Inhibited NLRP3 inflammasome activation and pyroptosis	[76]
	In vivo (*db/db* mice)	↓ ROS ↓ BMP4 via p38/JNK/caspase-3	Attenuates oxidative stress and vascular injury	[79]
	In vivo (Atherosclerotic mice)	↓ NF-ĸB ↓ NLRP3 inflammasome	Inhibits atherosclerotic lesion formation	[75]
p-Courmaric Acid	In vitro (Macrophage)	↓ NF-ĸB ↓ Foam cell formation ↓ Cytokine production	Protective effects against atherosclerosis	[81]
	In vivo (C57BL/6J)	↓ TLR4/NF-ĸB ↓ NLRP3 inflammasome ↓ ER stress ↓ Oxidative stress	Attenuated caspase-1 mediated IL-1β	[82]
Caffeic Acid	In vitro (HUVEC)	↑ NO bioavailability ↑ Endothelial proliferation ↑ Angiogenesis	Exhibits endothelial protective effects	[84]
	In vitro (LPS-induced HUVEC, CAME)	↑ HO-1/Nrf2 ↓ NF-ĸB ↓ COX-2 ↓ iNOS ↓ Cytokines	Anti-inflammatory and anti-allergic effects	[85]
	In vivo (*ApoE*^−/−)^	↑ ABCA1 ↑ ABCG1	Alleviates atherosclerosis via enhanced cholesterol efflux	[86]
	In vivo (Cerebral thrombosis model)	↓ Platelet adhesion and aggregation	Delays thrombus formation and vascular occlusion	[87]
Ferulic Acid	In vitro (HUVEC)	↑ ASS restored L-arginine-NO pathway ↓ NF-ĸB	Attenuates inflammation-driven endothelial dysfunction	[90]
	In vitro (HUVEC)	↓ p38 MAPK/NF-ĸB pathway	Reduced intracellular ROS, suppressed ICAM-1/VCAM-1, and attenuated NLRP3	[91]
	In vitro (VSMC)	↓ VSMC proliferation G1 arrest ↑ p21 via PI3K/Akt/eNOS	Suppresses VSMC phenotypic switching	[92]
	In vivo (*ApoE*^−/−)^	↑ AMPK ↓ SREBP1, ACC1, SCD1	Improves lipid metabolism and reduces plaque formation	[93]
**Hydrobenzoic Acid**				
**Compound**	**Experimental model**	**Mechanisms**	**Effects**	**Reference**
Gallic Acid	In vitro (macrophage)	↑ Nrf2 pathway	Suppressed ROS, inhibited NLRP3 and pyroptosis, may indirectly attenuate endothelial dysfunction	[95]
	In vivo (C57BL/6)	↓ Systolic blood pressure	Inhibited eNOS degradation, ROS generation, restored NO production, and mitigated endothelial dysfunction	[96]
	In vivo (*ApoE*^−/−)^	↓ Inflammation ↓ Plaque formation (male only)	Anti-atherosclerotic effects show sex-specific differences Low dose ineffective against atherogenesis	[97]
Vanillic Acid	In vitro (HUVEC)	↑ Akt-eNOS signaling ↑ NO ↓ Apoptosis	Restores endothelial function	[100]
	In vivo	↑ eNOS Normalized lipid metabolism	Mitigates NO-deficiency-driven vascular and metabolic dysfunction	[101]
Carnosic Acid	In vitro (Retinal EC)	↓ iNOS ↓ NO production ↓ Pro-inflammatory cytokine	Lower the damage of diabetic retinopathy	[104]
	In vivo (Rat)	↓ SIRT pathway ↓ ROS ↓ iNOS ↓ Cytokine	Protected retinal neuron damaged, preserved retinal microvascular and endothelial damage	[104]

## Data Availability

All data generated or analyzed during this study are included in this published article. The data that support the findings of this study are available from the corresponding author upon reasonable request.

## Abbreviations

ABCA1	ATP-binding cassette transporter A1
ABCG1	ATP-binding cassette transporter G1
ACC1	acetyl-CoA carboxylase 1
ACAT1	acetyl-CoA acetyltransferase 1
ACS	acute coronary syndrome
AGEs	advanced glycation end products
Akt	protein kinase B
AMPK	AMP-activated protein kinase
ASC	apoptosis-associated speck-like protein containing a CARD
ASS	argininosuccinate synthase
BMP4	bone morphogenetic protein 4
CaM	calmodulin
CAME	caffeic acid methyl ester
CD36	cluster of differentiation 36
CD99	cluster of differentiation 99
cGMP	cyclic guanosine monophosphate
COX-2	cyclooxygenase-2
CVD	cardiovascular disease
DAMP	damage-associated molecular pattern
EC	endothelial cell
EndMT	endothelial-to-mesenchymal transition
eNOS	endothelial nitric oxide synthase
EPC	endothelial progenitor cell
ER	endoplasmic reticulum
ERK1/2	extracellular signal-regulated kinase 1/2
FADD	Fas-associated death domain protein
FC	free cholesterol
FDA	U.S. Food and Drug Administration
FFA	free fatty acid
FOXO1	forkhead box O1
GSH-Px	glutathione peroxidase
HAEC	human aortic endothelial cell
HO-1	heme oxygenase-1
Hsp90	heat shock protein 90
HUVEC	human umbilical vein endothelial cell
ICAM-1	intercellular adhesion molecule-1
IL-1β	interleukin-1 beta
IL-18	interleukin-18
iNOS	inducible nitric oxide synthase
IP3R	inositol 1,4,5-trisphosphate receptor
JAM	junctional adhesion molecule
JNK	c-Jun N-terminal kinase
Keap1	Kelch-like ECH-associated protein 1
LAL	lysosomal acid lipase
LD	lipid droplet
LDL	low-density lipoprotein
LDL-C	low-density lipoprotein cholesterol
LDL-R	low-density lipoprotein receptor
LOX-1	lectin-like oxidized low-density lipoprotein receptor-1
LPS	lipopolysaccharide
MAPK	mitogen-activated protein kinase
MLCP	myosin light chain phosphatase
MyD88	myeloid differentiation primary response 88
NFAT	nuclear factor of activated T cells
NF-κB	nuclear factor kappa B
NLR	NOD-like receptor
NLRP3	NLR family pyrin domain containing 3
NO	nitric oxide
NOS	nitric oxide synthase
Nrf2	nuclear factor erythroid 2-related factor 2
oxLDL	oxidized low-density lipoprotein
PAI-1	plasminogen activator inhibitor-1
PAMP	pathogen-associated molecular pattern
PDGF-BB	platelet-derived growth factor-BB
PECAM-1	platelet endothelial cell adhesion molecule-1
PGI2	prostacyclin I2
PI3K	phosphoinositide 3-kinase
PKG	protein kinase G
PKM2	pyruvate kinase M2
PKR	protein kinase R
PL	phospholipid
PRR	pattern-recognition receptor
PSGL-1	P-selectin glycoprotein ligand-1
RIPK1	receptor-interacting serine/threonine-protein kinase 1
RNS	reactive nitrogen species
ROS	reactive oxygen species
SAA	salvianolic acid A
SAB	salvianolic acid B
SCD1	stearoyl-CoA desaturase 1
SERCA	sarco/endoplasmic reticulum calcium ATPase
SFI	salvianolate for injection
sGC	soluble guanylyl cyclase
SIRT1	sirtuin 1
SLC7A11	solute carrier family 7 member 11
SOD	superoxide dismutase
SR	sarcoplasmic reticulum
SR-A1	scavenger receptor class A member 1
SR-BI	scavenger receptor class B type I
SREBP1	sterol regulatory element-binding protein 1
TC	total cholesterol
TG	triglyceride
TLR	Toll-like receptor
TNF-α	tumor necrosis factor-alpha
TRIF	TIR-domain-containing adapter-inducing interferon-β
TXNIP	thioredoxin-interacting protein
VCAM-1	vascular cell adhesion molecule-1
VE-cadherin	vascular endothelial cadherin
VEGF	vascular endothelial growth factor
VSMC	vascular smooth muscle cell
